# Evaluation of oxidative stress markers in the heart and liver of rainbow trout (*Oncorhynchus mykiss* walbaum) exposed to the formalin

**DOI:** 10.1007/s10695-016-0260-0

**Published:** 2016-07-19

**Authors:** Halyna Tkachenko, Joanna Grudniewska

**Affiliations:** 1 Department of Zoology and Animal Physiology, Institute of Biology and Environmental Protection, Pomeranian University in Slupsk, Arciszewski Str. 22B, 76-200 Slupsk, Poland; 2 Department of Salmonid Research, Stanislaw Sakowicz Inland Fisheries Institute, Rutki, 83-330 Żukowo, Poland

**Keywords:** Rainbow trout *Oncorhynchus mykiss*, Formalin, Disinfection, Oxidative stress, Aminotransferases, Glycolytic potential

## Abstract

The aim of this study was to examine change in lipid and protein oxidation biomarkers, transamination enzymes and lactate dehydrogenase activities, lactate and pyruvate levels in liver and heart tissue of rainbow trout (*Oncorhynchus mykiss* Walbaum) that was exposed to formalin baths. Increase of 2-thiobarbituric acid reactive substances and carbonyl derivatives of protein oxidative destruction was noticed only in cardiac tissue of formalin-exposed fish. Activity of lactate dehydrogenase and lactate level in the cardiac tissue were elevated, indicating active glycolysis. Effects of formalin disinfection were different in both tissues. Aldehydic and ketonic derivatives of oxidatively modified proteins in liver were consistently reduced upon exposure to the formalin. In support of this, decrease in alanine and aspartate aminotransferases was noticed. Formalin disinfection of rainbow trout results in metabolic plasticity, predominantly in liver with decreased levels of oxidative stress biomarkers and aminotransferases activity. Formalin-induced oxidative stress in the cardiac tissue was more considerable.

## Introduction

Formalin is a generic term, which describes a solution of 37 % formaldehyde gas dissolved in water (Francis-Floyd 1996). It is used as a bath treatment to control external parasitic infections in fish (Bailey and Jeffrey 1989; Marking et al. 1994; Buchmann et al. 2004). Moreover, it is extremely effective against most protozoans, as well as some of the larger parasites such as monogenetic trematodes (Francis-Floyd 1996). It is not the preferred treatment for external bacterial or fungal infection. Formalin is not effective against internal infections of any type. It acts as a disinfectant, antiseptic, and astringent (FAO Fisheries and Aquaculture Dept. Aquaculture Management and Conservation Service 2007). Formalin has been effective in controlling fungal infection in rainbow trout; the agent effectively kills parasites on gills, skin, and fins (Bailey and Jeffrey 1989; Marking et al. 1994).

Small and Chatakondi (2006) have recommended three daily treatments of hybrid catfish (channel catfish *Ictalurus punctatus* × blue catfish *I. furcatus*) eggs with 100 ppm formalin as a 15-min bath. Eggs treated three times daily with 100 ppm formalin had the highest (*p* < 0.05) percentage of hatched eggs (Small and Chatakondi 2006). Barnes and Soupir (2007) have recommended either daily 15-min formalin treatments at concentrations of at least 750 mg/L or every-other-day treatments at 1667 mg/L to adequately control fungus and maximize rainbow trout egg survival. Wagner et al. (2008) have compared the bactericidal ability of four common disinfectants (formalin, iodine, rock salt, and hydrogen peroxide) in vivo on rainbow trout eggs. Formalin and hydrogen peroxide reduced bacterial abundance but were inferior to iodine in some cases. A treatment of 1667 mg of formalin per L of water significantly reduced bacterial abundance (Wagner et al. 2008). Lahnsteiner and Kletzl (2016) have recommended use of formalin at a concentration of up to 1500 ppm to disinfect pikeperch (*Sander lucioperca*) eggs. Embryos in the morula stage, epiboly stage, and at the beginning of heart beat and blood circulation tolerated formalin concentrations of up to 1500 ppm for 15 min (Lahnsteiner and Kletzl 2016).

Although formalin may continue to be useful in the aquaculture industry, it induces potentially harmful alterations to fish skin (Sanchez et al. 1998) and bronchial lesions (Speare et al. 1997). It was reported that exposure of rainbow trout to various concentrations of formalin affected the mucous cells indicated by increased release of mucus (Buchmann et al. 2004). Blabbing of epithelial cell membranes was the first sign of the injury. Highly irregular organization of cells followed, with regional differences occurring in different parts of fins (Buchmann et al. 2004). Formalin is a potential carcinogen and should be handled with care to avoid skin contact, eye irritation, and inhalation (Thrasher and Kilburn 2001). Previous study indicated that formaldehyde exposure could lead to inflammation and in consequence to oxidative stress (Saito et al. 2005; Persoz et al. 2010).

Formaldehyde can induce oxidative stress by increasing the formation of reactive oxygen species (ROS) (Saito et al. 2005; Bono et al. 2010; Szende and Tyihák 2010). Formaldehyde intoxication may stimulate oxidative stress and thus, some secondary toxic effects in cardiac cells and tissue (Güleç et al. 2006). ROS formation in rats incubated with low concentration of formaldehyde was indicated by decrease in mitochondrial membrane potential and inhibition of mitochondrial respiration. All of the changes were dose dependent and measured in isolated hepatocytes (Teng et al. 2001). Moreover, the toxicity of formaldehyde has been attributed to its ability to form adducts with DNA and proteins (Teng et al. 2001). On the other hand, formaldehyde covalently binds with proteins to form formaldehyde-protein conjugates, which may lead to the formation of formaldehyde-specific antibodies (Li et al. 2007).

ROS can interact with DNA and lipids in one of the ways including activation of oxidases and the inhibition of scavenger systems, leading to oxidative damage and lipid peroxidation (Li et al. 2007; Bono et al. 2010). During lipid peroxidation, malonic dialdehyde (MDA), a highly reactive dialdehyde, can be generated (Gaté et al. 1999). MDA can react with the free amino group of proteins, phospholipids or nucleic acids, to produce inter- and intramolecular 1-amino-3-iminopropene bridges and structural modifications of biological molecules (Halliwell and Gutteridge 1989; Halliwell 1994). Proteins are targets for free radicals (Shacter 2000). Protein oxidation is defined as the covalent modification of a protein induced either directly by ROS or indirectly by reaction with secondary by-products of oxidative stress leading to the modification of certain amino acid residues, forming carbonyl derivatives (Shacter 2000). Protein oxidation may be partially responsible for alterations of signal transduction mechanisms, transport systems, or enzyme activities (Gaté et al. 1999). Formaldehyde reacts chemically with organic compounds (e.g., deoxyribonucleic acid, nucleosides, nucleotides, proteins, amino acids) by addition and condensation reactions, thus forming adducts and deoxyribonucleic acid-protein cross-links (Thrasher and Kilburn 2001).

The aminotransferases are the most specific indicators of cellular necrosis (Thapa and Walia 2007). Aspartate aminotransferase (AST) and alanine aminotransferase (ALT) catalyze the transfer of the amino acids of aspartate and alanine, respectively, to the ketogroup of ketoglutaric acid. ALT is primarily localized in the liver but the AST is present in a wide variety of tissues (heart, skeletal muscle, kidney, brain, and liver) (Friedman et al. 2003; Thapa and Walia 2007). Lactate dehydrogenase (LDH), an intracellular enzyme constitutes a major checkpoint of anaerobic glycolysis, by catalyzing the conversion of lactate to pyruvate and indicates cellular damage (Granchi et al. 2010). The activities of transaminases and LDH are relevant stress indicators (Thapa and Walia 2007). A significant change in the activity of these enzymes indicates amplified transaminases processes and stress-induced tissue impairment (Granchi et al. 2010).

Rainbow trout (*Oncorhynchus mykiss* Walbaum) is a good model organism for toxicological research and responses to various xenobiotics and environmental contaminants (Gomez et al. 1997; Thorgaard et al. 2002; Carvan et al. 2008; Escher et al. 2011; Li et al. 2011; Williams 2012). Moreover, it is a prominent model for studies involving carcinogenesis, comparative immunology, and physiology (Thorgaard et al. 2002). Since naturally reproducing populations of rainbow trout appear on several continents, this species serves as an ecotoxicogenomic model, bridging the gap between the laboratory and natural aquatic environments (Carvan et al. 2008). Therefore, the objective of this study was to examine the impact of formalin-induced disinfection on level of oxidative stress biomarkers (2-thiobarbituric acid reactive substances as lipid peroxidation biomarker, aldehydic and ketonic derivatives as biomarkers of oxidatively protein damage), as well as biomarkers of aerobic-anaerobic metabolism (aminotransferases and lactate dehydrogenase activities, lactate and pyruvate contents) in the hepatic and cardiac tissues of rainbow trout.

## Materials and methods

### Experimental fish

Clinically, healthy rainbow trout (*n* = 21) with a mean body mass of (45.0 ± 2.2) g was used in the experiments. The study was carried out in a Department of Salmonid Research, Inland Fisheries Institute in Rutki, Poland. Experiments were performed at a water temperature of 16 ± 2 °C, and the pH was 7.5. The dissolved oxygen level was about 12 ppm with additional oxygen supply with a water flow of 25 L per min and a photoperiod of 7 h per day. The fish were fed with commercial pelleted diet. All enzymatic assays were carried out at Department of Zoology and Animal Physiology, Institute of Biology and Environmental Protection, Pomeranian University in Słupsk (Poland).

### Experimental groups

The fish were divided into two groups and held in 250-L square tanks (70 fish per tank) supplied with the same water as during the acclimation period (2 days). On alternative days, water supply to each tank was stopped. Fish were disinfected with formalin in final concentration 200 mL per m^3^ (Group II, *n* = 10). For short-term baths, a concentration of 250 mg per L can be delivered for 30 to 60 min. At moderate water temperatures (less than 21 °C), fish can be left in a 250 mg per L formalin bath for about 1 hour. At warmer water temperatures (greater than 21 °C), a concentration of formalin should be decreased to 150 mL per L for no more than one hour (Francis-Floyd 1996). Control fish (Group I, *n* = 11) were handled in the same way as formalin-exposed group with the same water from square tanks. Rainbow trout was exposed to formalin three times, once a day with 20-min exposure every 3 days (1st, 4th, and 7th days of the experiment). Two days after the last bathing fish were sampled. Fish were not anesthetized before tissue sampling.

### Tissue isolation

Hearts and livers were removed from trout after decapitation. One trout was used for each homogenate preparation. Briefly, the liver and heart from each fish were excised, weighted, and washed in ice-cold Tris–HCl buffer. The organs were rinsed clear of blood with cold isolation buffer and homogenized in a glass Potter–Elvehjem homogenizing vessel with a motor-driven Teflon pestle on ice in proportion 1:9 (weight/volume). The isolation buffer contained 100 mM Tris–HCl; a pH of 7.2 was adjusted with HCl. Homogenates were centrifuged at 3000*g* for 15 min at 4 °C. After centrifugation, the supernatant was collected and frozen at −20 °C until analyzed. One heart and one liver from each fish were used in biochemical assays. All enzymatic assays were carried out at 25 ± 0.5 °C using a Specol 11 spectrophotometer (Carl Zeiss Jena, Germany). Adding the homogenate suspension started the enzymatic reactions. The specific assay conditions are presented subsequently. Each sample was analyzed three times. The protein concentration in each sample was determined according to Bradford (1976) using bovine serum albumin as a standard.

### Oxidative stress biomarkers assay

#### Assay of TBARS level

An aliquot of the homogenate was used to determine the lipid peroxidation status of the sample by measuring the concentration of 2-thiobarbituric acid-reacting substances (TBARS), according to the method of Kamyshnikov (2004). Reaction mixture contained sample homogenate (2.1 mL, 10 % w/v) in Tris–HCl buffer (100 mM, pH 7.2), 2-thiobarbituric acid (TBA; 0.8 %, 1.0 mL), and trichloracetic acid (TCA; 20 %, 1.0 mL). The total volume was kept in a water bath at 100 °C for 10 min. After cooling, mixture was centrifuged at 3000*g* for 10 min. The absorbance of the supernatant was measured at 540 nm. TBARS values were reported as nmoles malonic dialdehyde (MDA) per mg protein.

#### Assay of carbonyl groups of oxidatively modified proteins (OMP) level

Carbonyl groups were measured as an indication of oxidative damage to proteins according to the method of Levine et al. (1990) in modification of Dubinina et al. (1995). Samples were incubated at room temperature for 1 h with 10 mM 2,4-dinitrophenylhydrazine (DNTP) in 2 M HCl. Blanks were run without DNTP. Afterward, proteins were precipitated with TCA and centrifuged for 20 min at 3000*g*. The protein pellet was washed three times with ethanol/ethylacetate (1:1) and incubated at 37 °C until complete resuspension. The carbonyl content could be measured spectrophotometrically at 370 nm (aldehydic derivatives, OMP_370_) and at 430 nm (ketonic derivatives, OMP_430_) (molar extinction coefficient 22,000 M^−1^ cm^−1^) and expressed as nmol per mg protein.

#### Assays of ALT (E.C. 2.6.1.2) and AST (E.C. 2.6.1.1) activities

ALT and AST activity was analyzed spectrophotometrically by standard enzymatic method (Reitman and Frankel 1957). The ketoacids produced by the enzyme action reacts with 2,4-dinitrophenylhydrazine producing hydrazone complex measured calorimetrically at 530 nm. ALT and AST activities were expressed as μmol pyruvate per h per mg of protein.

#### Assay of LDH (E.C. 1.1.1.27) activity

The colorimetric method of Sevela and Tovarek (1959) was used for the determination of LDH activity LDH activity was expressed as mmol pyruvate per h per L of blood.

#### Assays of lactate and pyruvate concentrations

Lactate and pyruvate concentration was measured according to the procedure described by Herasimov and Plaksina (2000). One mL of tissue homogenate sample was added to 6 mL distilled water and 1 mL metaphosphoric acid (10 %). The mixture was centrifuged at 800*g* for 5 min to separate the supernatant. 1 mL CuSO_4_ (25 %) and 0.5 g Ca(OH)_2_ were added to the supernatant, which was then mixed for 30 min. The mixture was centrifuged at 1000*g* for 10 min. For lactate concentration assay, the resulting supernatant was resuspended in 3 mL *p*-dimethylamino benzaldehyde and 1 mL NaOH (25 %). Solutions were heated in a water bath at 37 °C for 45 min, which was then centrifuged at 1000*g* for 10 min. The absorbance was measured at 420 nm. Solution with *p*-dimethylamino benzaldehyde and NaOH (25 %) was used as blank. For pyruvate concentration assay, the resulting supernatant was resuspended in 0.1 mL CuSO_4_ (10 %), 4 mL H_2_SO_4_, and 0.1 mL hydroquinone, which was then heated in a water bath at 100 °C for 15 min. The absorbance was measured at 430 nm. Calibration curve of lactate (0.1–5 mM) and pyruvate (0.1–5 mM) was used, and results were expressed in nmol per mg protein.

### Statistical analysis

Results are expressed as mean ± S.E.M. All variables were tested for normal distribution using the Kolmogorov–Smirnov test (*p* > 0.05). Significance of differences in the oxidative stress biomarkers in the heart and liver of rainbow trout between control and formalin-exposed groups (significance level at *p* < 0.05) was examined using Mann–Whitney *U* test according to Zar (1999). In addition, the relationships between oxidative stress biomarkers of all individuals were evaluated using Spearman’s correlation analysis. Statistical calculation was performed on separate data from each individual with STATISTICA 8.0 (StatSoft, Poland).

## Results

**Fig. 1:**
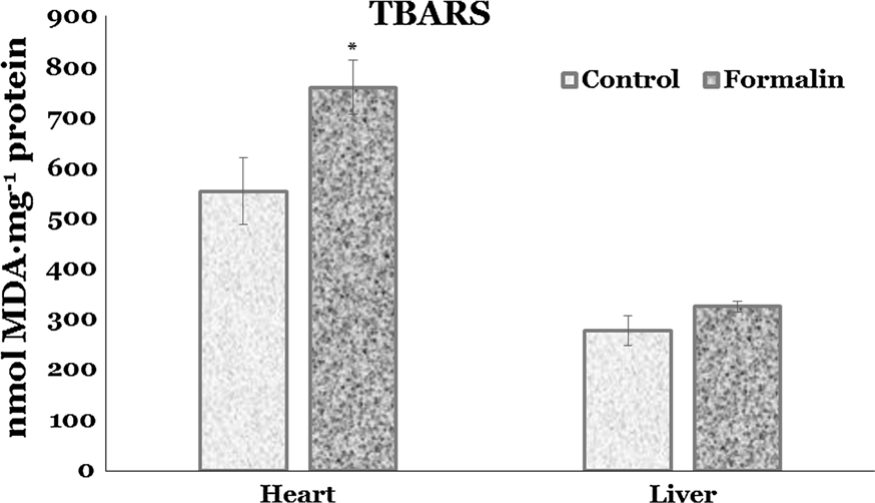
Lipid peroxidation measured by quantity of TBARS level in the heart and liver of rainbow trout exposed to formalin disinfection. Values expressed as mean ± S.E.M. *Asterisk* the significant change was shown as *p* < 0.05 when compared to untreated group values

The results in Fig. 1 indicate that the trout exposed to formalin expressed a significantly higher TBARS level in the cardiac tissue by 37.2 % (*p* = 0.020) compared to untreated group. No significant differences in lipid peroxidation in the liver between control and formalin-exposed group were found (Fig. 1).

**Fig. 2:**
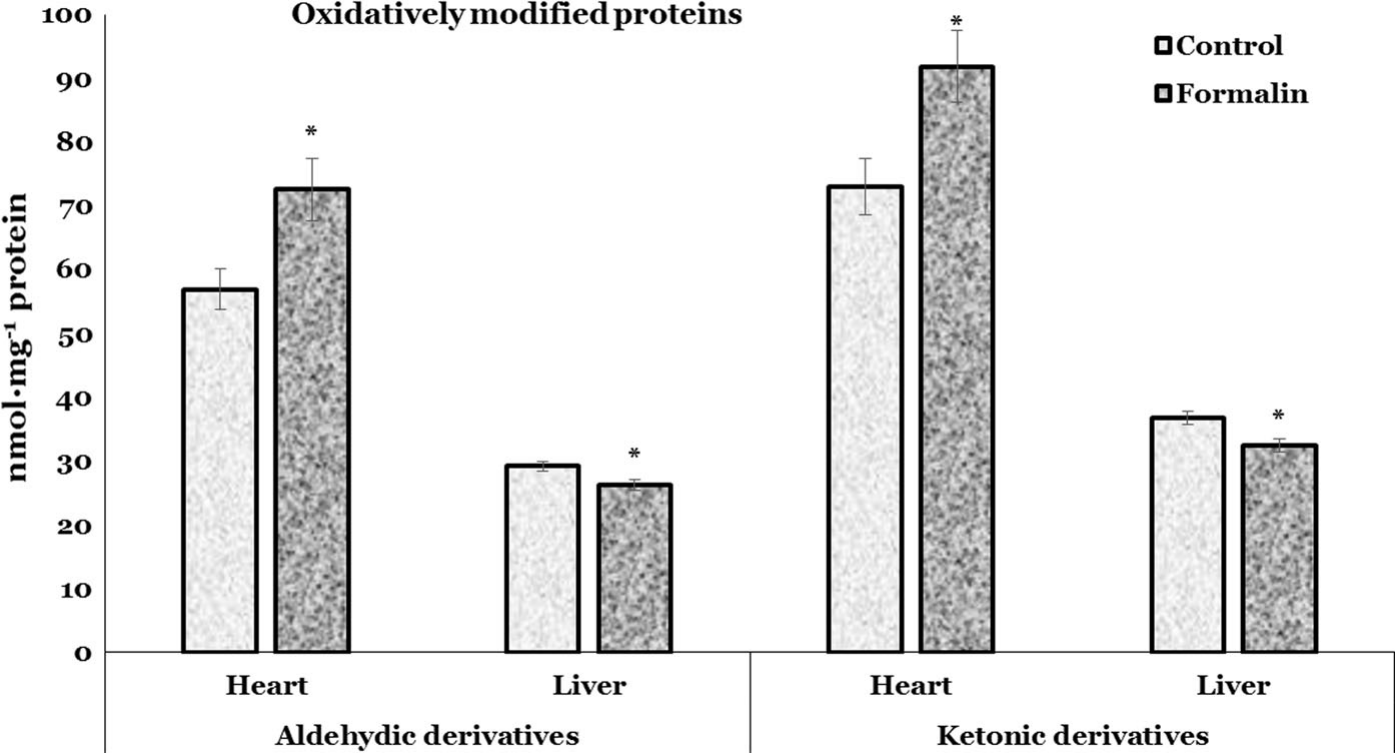
Level of the aldehydic and ketonic derivatives of OMP in the heart and liver of rainbow trout exposed to formalin. Values expressed as mean ± S.E.M. *Asterisk* see Fig. 1

Figure 2 shows the level of aldehydic and ketonic derivatives of OMP in the cardiac and hepatic tissues of formalin-exposed trout. Levels of aldehydic and ketonic derivatives of OMP were significantly lower (by 9.9 %, *p* = 0.020 and by 12 %, *p* = 0.014, respectively) in the liver of formalin-exposed trout compared to control group. In contrast, the aldehydic and ketonic derivatives of OMP in the heart of formalin-exposed group were significantly higher than in control group by 27 % (*p* = 0.024) and 26 % (*p* = 0.035), respectively (Fig. 2).

**Fig. 3:**
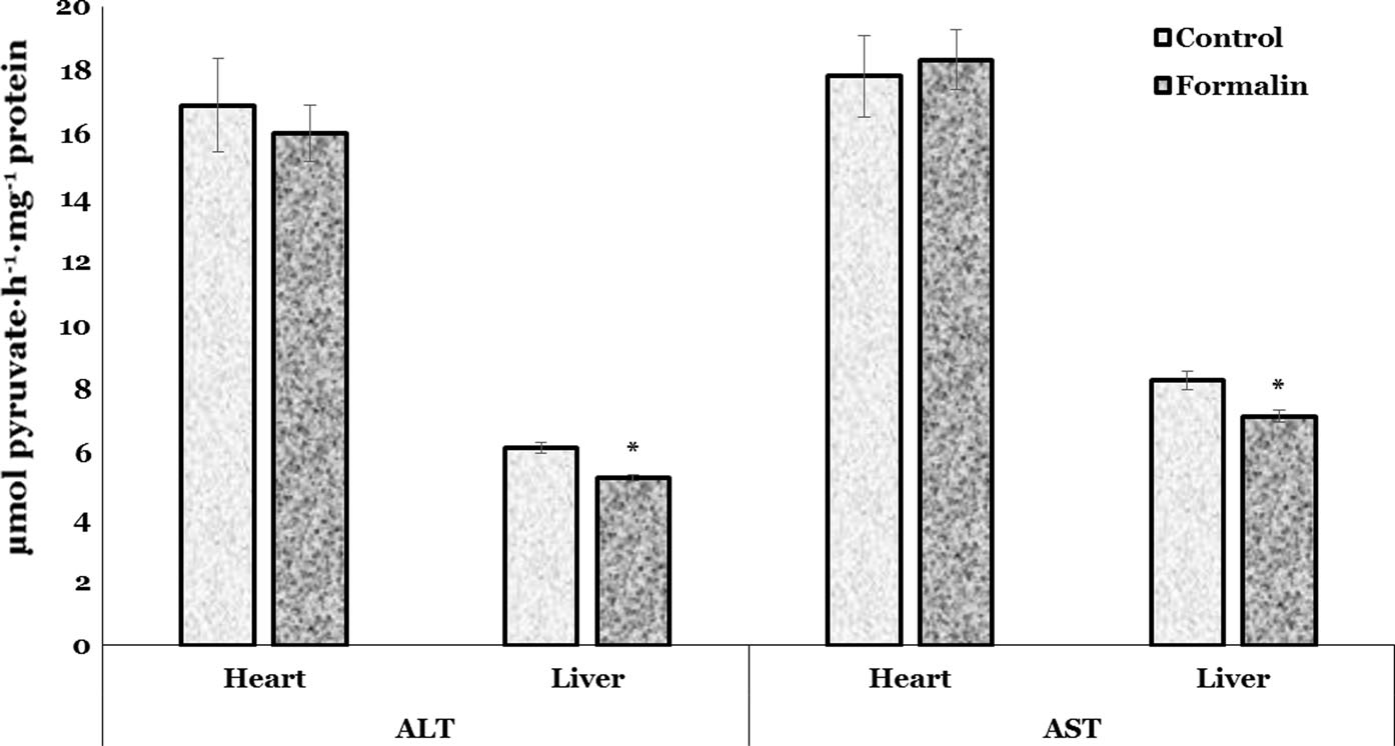
ALT and AST activities in the heart and liver of rainbow trout exposed to formalin disinfection. Values expressed as mean ± S.E.M. *Asterisk* see Fig. 1

ALT and AST activities are used as indicators of cell damage (Thapa and Walia 2007). Hepatic biomarkers (ALT and AST activities) according to Fig. 3 were significantly decreased in the liver of the formalin-exposed trout by 15.3 % (*p* = 0.000) and 13.5 % (*p* = 0.004), respectively, as compared with control group. Fig. 3 also revealed that ALT and AST activities in the cardiac tissue of formalin-exposed trout were nonsignificantly altered.

**Fig. 4:**
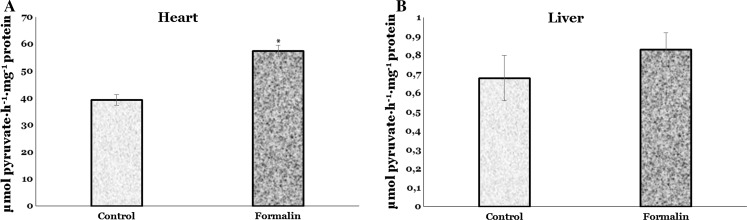
LDH activity in the heart and liver of rainbow trout exposed to formalin disinfection. Values expressed as mean ± S.E.M. *Asterisk* see Fig. 1

As shown in Fig. 4a, LDH activity was significantly higher only in the cardiac tissue of formalin-exposed trout than that in untreated fish (by 46 %, *p* = 0.000). In hepatic tissue, a nonsignificant increase (by 22 %, *p* > 0.05) was noted (Fig. 4b).

**Fig. 5:**
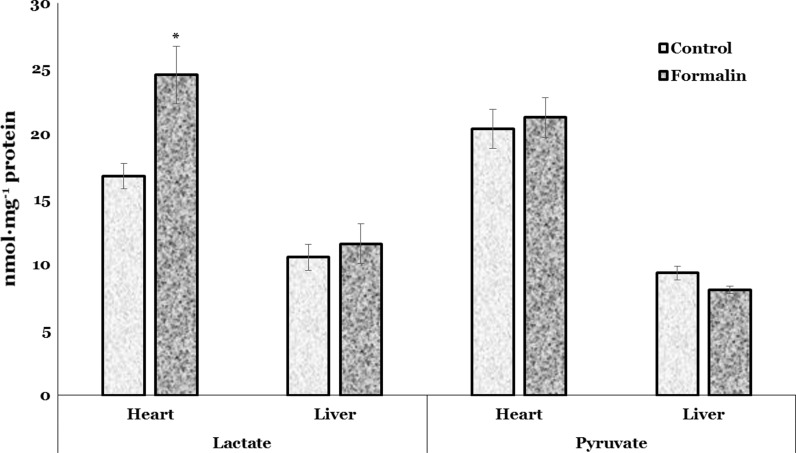
Lactate and pyruvate levels in the heart and liver of rainbow trout exposed to formalin disinfection. Values expressed as mean ± S.E.M. *Asterisk* see Fig. 1

Figure 5 represents the lactate and pyruvate concentrations in the liver and heart of formalin-exposed trout. Regarding the lactate, its level was increased by 46 % (*p* = 0.003) only in the heart of formalin-exposed trout compared to values of untreated fish. Pyruvate level was nonsignificantly altered in the heart and liver of disinfectant-treated trout (Fig. 5).

**Fig. 6:**
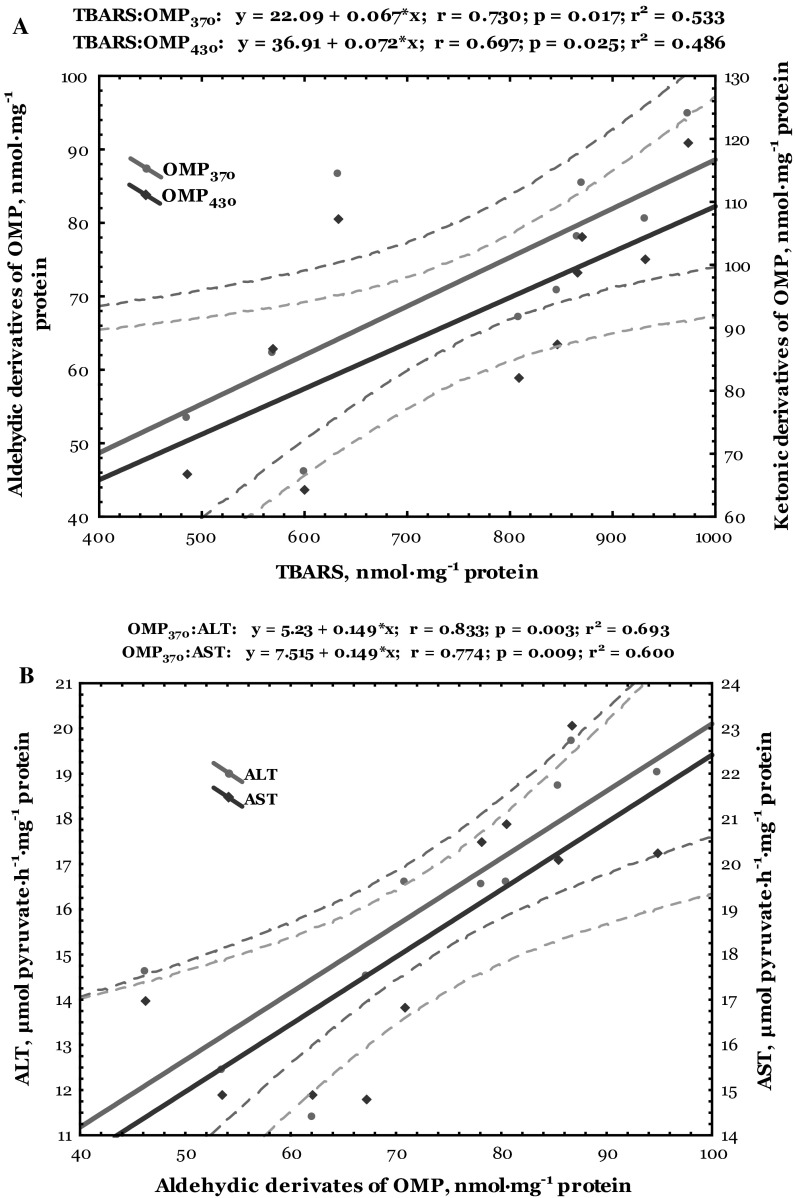

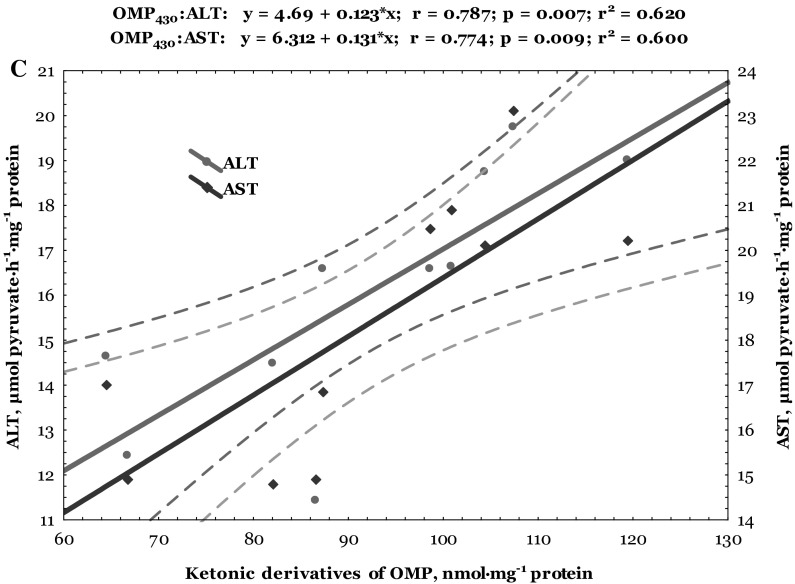
Correlations between levels of TBARS, aldehydic and ketonic derivatives of oxidatively modified proteins (**a**), as well as between ALT and AST activities and aldehydic (**b**) and ketonic derivatives (**c**) in the heart of trout disinfected by formalin

**Fig. 7:**
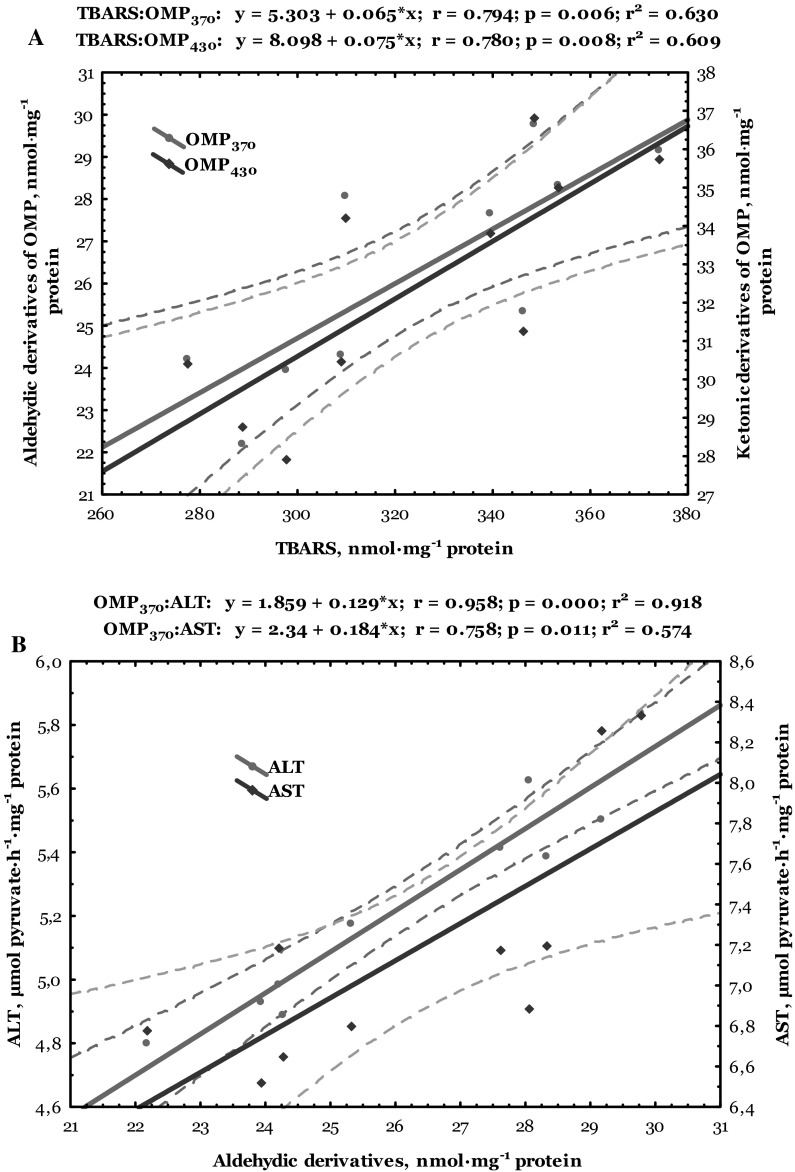

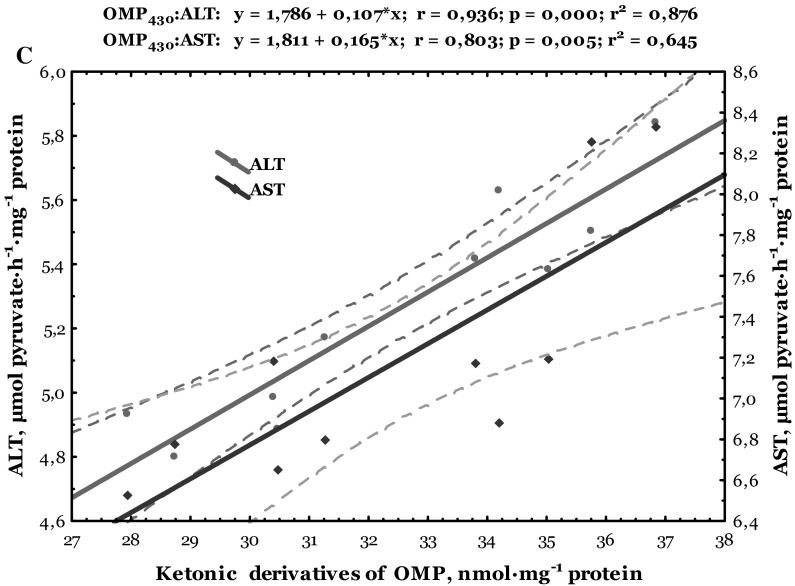
Correlations between levels of TBARS, aldehydic and ketonic derivatives of oxidatively modified proteins (**a**), as well as between ALT and AST activities and aldehydic (**b**) and ketonic derivatives (**c**) in the liver of trout disinfected by formalin

Data of correlative analysis between lipid peroxidation, oxidative modified protein levels, and biochemical enzymes activity in the heart of trout disinfected by formalin are shown in Figs. 6 and 7. Both cardiac and hepatic TBARS level correlated positively with aldehydic (*r* = 0.730, *p* = 0.017 and *r* = 0.794, *p* = 0.006, respectively) and ketonic derivatives of oxidatively modified proteins (*r* = 0.697, *p* = 0.025 and *r* = 0.780, *p* = 0.008, respectively) (Figs. 6a, 7a). Synergism between aldehydic derivatives of oxidatively modified proteins and ALT (*r* = 0.833, *p* = 0.003 and *r* = 0.958, *p* = 0.000, respectively) and AST activities in the cardiac and hepatic tissues (*r* = 0.774, *p* = 0.009 and *r* = 0.758, *p* = 0.011, respectively) was also verified (Figs. 6b, 7b). Also, the ketonic derivatives of OMP were positively correlated with ALT (*r* = 0.787, *p* = 0.007 and *r* = 0.936, *p* = 0.000, respectively) and AST activities (*r* = 0.774, *p* = 0.009 and *r* = 0.803, *p* = 0.005, respectively) in the heart and liver of trout disinfected by formalin (Figs. 6c, 7c).

## Discussion

ROS-mediated oxidative damage could play an important role in formaldehyde toxicity and has been detected in various cells and tissues (Saito et al. 2005; Bono et al. 2010; Szende and Tyihák 2010). Our results indicate changes in lipid peroxidation biomarker (TBARS level) only in the cardiac tissue of formalin-exposed trout. Results of this study showed that formalin caused oxidative stress (increased lipid peroxidation and oxidatively modification of proteins) evidenced by alteration of non-specific biochemical indicators of organopathies given as increased LDH activity and lactate concentration in the cardiac tissue (Figs. 1, 2, 3, 4, 5). Spearman’s correlative analysis also indicated that aldehydic and ketonic derivatives of protein oxidation were influenced by ALT and AST activities (Figs. 6b, c). Direct relationships between lipid peroxidation (TBARS level), ketonic and aldehydic derivatives of OMP, as well as between aminotransferases activity both in the cardiac and hepatic tissues of formalin-exposed trout were noted (Figs. 6, 7). We conclude that oxidatively modified protein contents, lipid peroxidation, LDH activity, and lactate concentration may function as useful biomarkers for formalin-induced oxidative stress only in cardiac tissue.

Our results are in agreement with reports from other researchers, who suggest that formaldehyde can induce oxidative stress by increasing the formation of ROS (Saito et al. 2005; Güleç et al. 2006; Bono et al. 2010; Szende and Tyihák 2010). Saito et al. (2005) assessed two kinds of oxidative stress markers: cellular glutathione (GSH) content and cellular ROS, as well as the DNA–protein cross-links, which formed as the result of formaldehyde treatment using Jurkat cells. A marked decrease in total cellular GSH, increase of cellular ROS before cell death, formation of DNA–protein cross-links in the presence of formaldehyde were observed (Saito et al. 2005). TBARS, a lipid peroxidation biomarker commonly used as an indicator of oxidative damage, was significantly higher in the cardiac tissues of male rats exposed to formaldehyde in subacute and subchronic studies (Güleç et al. 2006). Teng et al. (2001) observed formation of ROS in isolated rat hepatocytes incubating with low concentrations of formaldehyde (2001). Moreover, formaldehyde may exert these oxidative stress effects in tissues indirectly, mediated by an inflammatory response (Saito et al. 2005; Persoz et al. 2010). The reaction of formaldehyde with amino groups of proteins is critical in inducing an immune response in vivo (Li et al. 2007). Yildiz et al. (2009) found that non-specific immune parameters of rainbow trout after exposure to formalin have undergone alterations in general. The increase in hematocrit, leucocrit, and serum glucose levels in fish exposed to formalin was noted (Yildiz et al. 2009). Im et al. (2006) investigated the effects of formaldehyde on rat plasma proteins. Proteins involved in apoptosis, transportation, signaling, energy metabolism, cell structure, and motility were found to be up- or down-regulated dependant on formaldehyde exposure (Im et al. 2006). Cytotoxic effects of formaldehyde in rat lung tissues exposed to ambient air and two different concentrations of formaldehyde (0, 5, 10 ppm) for 2 weeks at 6 h/day and 5 days/week in an inhalation chamber were confirmed by Sul et al. (2007).

Formaldehyde can induce oxidative stress. It is responsible for increase in ROS formation in many tissues. ROS can interact with DNA and lipids, leading to oxidative damage and lipid peroxidation, respectively (Gurel et al. 2005; Saito et al. 2005; Kum et al. 2007). MDA is a natural product of lipid peroxidation that can react with DNA to form exocyclic adducts, including the 3-(2-deoxy-β-D-erythro-pentafuranosyl)pyrimido[1,2-α]purin-10(3H)-one dG (M1dG), that, if not repaired, can induce base pair mutations and cause frame-shift mutations in reiterated sequences (Marnett 2002; VandervVeen et al. 2003). Previous studies have also shown that the formation of M1dG adducts could be associated to increased cancer risk and tumor progression (Munnia et al. 2004, 2006). M1dG adduct measurement is considered to be a biomarker that reflects air pollutant exposure capable to induce oxidative stress and ROS (Peluso et al. 2010). Bono et al. (2010) analyzed the effect of formaldehyde exposure on leukocyte malondialdehyde-deoxyguanosine adducts (M1dG), a biomarker of oxidative stress and lipid peroxidation in a group of Italian pathologists. Working in the reduction rooms and exposure to air-formaldehyde concentrations higher than 66 µg/m^3^ are associated with increased levels of M1dG adducts (Bono et al. 2010).

Moreover, formaldehyde-induced toxic damage involves ROS that trigger subsequent toxic effects and inflammatory responses. Results of Murta et al. (2016) point out to the potential of formaldehyde in promoting airway injury by increasing the inflammatory process as well as by the redox imbalance [an increase of macrophages and lymphocytes, NADPH oxidase in the blood, activity of superoxide dismutase (SOD) and catalase, total glutathione (tGSH), reduced glutathione (GSH) and oxidized glutathione (GSSG), an increase in lipid peroxidation and CCl_2_, CCl_3_ and CCl_5_ chemokines, as well as decrease of the reduced/oxidized glutathione ratio (GSH/GSSG)]. Yu et al. (2014) have suggested that a certain concentration of formaldehyde (20, 40, 80 mg/m^3^) for 15 days in the respective inhalation chambers could have toxic effects on the hematopoietic system, with oxidative stress as a critical effect. SOD activity, mitochondrial membrane potential, and Bcl-2 expression were decreased with increasing formaldehyde concentration, while expression of Bax and cytochrome C and MDA content were increased (Yu et al. 2014).

Moreover, formaldehyde, a cytotoxicant at high doses, induces leukemia. Heck and Casanova (1999) have suggested that formaldehyde-induced DNA–protein cross-links are genotoxic as a result of their ability to arrest DNA replication. Although they can be removed and the DNA can be repaired, failure to remove the blockage prior to cell division or excision followed by incomplete repair could cause cell death or a mutation. The arrest of DNA replication at high formaldehyde concentrations could result in cytolethality or genotoxicity, both of which are critical factors in the induction of rat nasal cancer by formaldehyde. However, at concentrations below 2 ppm in monkeys or 1 ppm in rats, the decrease in the rate of DNA replication is predicted to be <1 % after a 6-h exposure (Heck and Casanova 1999). Santovito et al. (2011) have evaluated the frequency of chromosomal aberrations in peripheral blood lymphocytes from workers in pathology wards who have been exposed to formaldehyde compared with a group of unexposed subjects. Air formaldehyde induces chromosomal aberrations even consequently to low levels of daily exposure, indicating an increased risk of genetic damage for workers exposed to this air pollutant (Santovito et al. 2011).

Exposure to formaldehyde causes irritation of the respiratory mucosa and is associated with inflammation and oxidative stress in the airways. Lima et al. (2015) have studied the oxidative effects on the inflammatory response in the trachea and the diaphragm muscle of rats exposed to different concentrations of formaldehyde (1, 5, 10 %). The exposure to formaldehyde at different concentrations in a short period of time promotes oxidative damage and inflammation in the diaphragm muscle and the trachea and causes metaplasia, ulceration, and increased mucus at the latter. There was an increase of lipid and protein peroxidation and decrease of catalase in the trachea and the diaphragm muscle. In formaldehyde group, the tracheal epithelium showed metaplasia and ulceration (Lima et al. 2015).

In addition, dose-related induction of MDA production in plasma and liver was demonstrated in experiments carried out in vivo on rats exposed to the agent (Im et al. 2006; Kum et al. 2007). The study of Attia and co-workers (2016) revealed significant increase in the levels of formate, MDA, and p53 as a biomarker of carcinogenesis among workers exposed to formaldehyde in cosmetic industry compared with their control group. The ROS and lipid peroxidation are involved in formaldehyde-induced genotoxicity in human lung cancer cell lines A549 (Zhang et al. 2013). Formaldehyde-induced genotoxicity through its ROS and lipid peroxidase activity and caused DPCs effects in A549 cells. Formaldehyde exposure caused induction of DNA–protein cross-links (DPCs). Formaldehyde significantly increased MDA levels, and decreased SOD and glutathione peroxidase (GSH-Px) activity. In addition, the activation of NF-κB and AP-1 was induced by formaldehyde treatment (Zhang et al. 2013). Shi et al. (2014) have hypothesized that ROS and lipid peroxidation are involved in formaldehyde-induced genotoxicity in human lung cancer cell line, A549 cell line. The results indicated that exposure to formaldehyde showed the induction of DNA–protein cross-links. Formaldehyde significantly increased the malondialdehyde levels and decreased the activities of superoxide dismutase and glutathione peroxidase. In addition, the activation of necrosis factor-κB (NF-κB) and activator protein 1 (AP-1) was induced by the formaldehyde treatment (Shi et al. 2014).

Lipid peroxidation break down products such as hydroxynonenal, MDA, and acrolein bind covalently to histidine, lysine, cysteine residues, leading to the addition of aldehyde moieties to the protein (Shacter 2000). Oxidative modification of enzymes can have either mild or severe effect on cellular or systemic metabolism, depending on the percentage of molecules that are modified and the chronicity of the modification (Shacter 2000). According to our results, it can be considered that exposure of rainbow trout to formalin caused oxidative modification of proteins and indicated increase of oxidative protein destruction (Fig. 2), as well as lipid peroxidation in the cardiac tissue (Fig. 1). Moreover, our results emphasize the significant impact of formalin-induced oxidative stress in the cardiac tissue on the biochemical activity of important enzymes, especially on significantly increased LDH activity and lactate level (Figs. 4, 5). Transaminases activities used as indicators of cell damage (Thapa and Walia 2007) play an important role in protein and amino acid metabolism both in the cardiac and hepatic tissues of formalin-exposed trout (Figs. 6, 7). The activity of transaminases in fish may be significantly changed under the influence of different toxic agents (Zikić et al. 2001). Oxidative stress caused by different xenobiotics may damage certain tissues and liberate various transaminases into the plasma (Zikić et al. 2001).

In our study, we have shown that cardiac function was particularly sensitive to formalin exposure in rainbow trout during disinfection. The mode of action of formalin may involve a direct impact on enzyme activities or indirect by impinging on the tissue energy budget of the animal. The indirect effect of formalin on metabolic capacity is supported by the significant elevation of TBARS and carbonyl contents of protein oxidation (Figs. 1, 2) and positive correlation between both aldehydic and ketonic derivatives and aminotransferases activities (Fig. 6b, c), suggesting a greater cardiac damage in response to formalin-induced oxidative stress.

Higher cardiac LDH activity coupled with elevated lactate level in formalin-treated trout suggests an enhanced glycolytic potential, providing ATP for cardiac function (Figs. 4, 5). Therefore, it is likely that this process is involved in enhancement of aldehydic and ketonic derivatives of oxidatively modified proteins (Fig. 2). While LDH activity (an indicator of glycolysis capacity) showed no significant change in hepatic tissue of formalin-exposed trout, the decrease in ALT and AST activities (a key enzymes involved in amino acid catabolism and providing C3 substrates for gluconeogenesis) supports a decreased liver gluconeogenic capacity. The activities of transaminases are thought to be a more reliable measure of tissue gluconeogenic capacity in teleosts (Mommsen et al. 1999; Vijayan et al. 2003). Maintenance of liver glycogen content, albeit at a reduced level, by altered liver capacity for gluconeogenesis appears to be important adaptive strategies during disinfection by formalin and may be regulated by aldehydic and ketonic derivatives of OMP (Fig. 7b, c).

Also, the significantly depressed liver aminotransferases activity, without any associated changes in lactate and pyruvate concentrations (Figs. 3, 4) supports decreased in amino acid catabolism that may be related to the formalin exposure. Consequently, the decreased tissue metabolic demands, including ATP requirements for synthesis of proteins, in the liver of formalin-treated trout may lead to suppression of oxidative stress including protein oxidation (Fig. 2).

Liver ALT and AST activities were inhibited by formalin exposure, leading to the proposal that aminotransferases are more sensitive than LDH to formalin disinfection in this species. As amino acids, especially alanine, are preferred substrates for gluconeogenesis in fish (Mommsen et al. 1999), the lower ALT activity with formalin implicates a reduced liver capacity for amino acid catabolism and gluconeogenesis. As gluconeogenesis is playing a key role in maintaining hyperglycemia, the depression in the liver gluconeogenic capacity with formalin may be a key factor in the reduced liver carbonyl contents of OMP observed in the formalin-exposed group (Figs. 2, 3). This reduction in liver capacity for gluconeogenesis may also be contributing to the lower glycogen levels, especially since the non-altered liver LDH activity with formalin disinfection argue against glycolysis in the hepatic tissue.

Formalin disinfection of rainbow trout results in metabolic plasticity, predominantly in liver with decreased levels of oxidative stress biomarkers and aminotransferases activity, while increased level of aldehydic and ketonic derivatives of oxidatively modified proteins and lipid peroxidation, as well as lactate dehydrogenase and lactate level in the cardiac tissue was observed. On the other hand, disinfection of trout by formalin in dose 200 mL per m^3^ caused oxidative stress in the cardiac tissue. Understanding the role of biochemical changes in the tissues of formalin-exposed trout has important implications for understanding of the complex physiological changes that occur during disinfection but also for improving aquaculture practices to maximize tissues growth and health of treated trout.

## References

[CR1] Bailey TA, Jeffrey M (1989). Evaluation of 215 candidate fungicides for use in fish culture.

[CR2] Barnes MA, Soupir CA (2007). Evaluation of formalin and hydrogen peroxide treatment regimes on rainbow trout eyed eggs. N Am J Aquac.

[CR3] Bono R, Romanazzi V, Munnia A, Piro S, Allione A, Ricceri F, Guarrera S, Pignata C, Matullo G, Wang P, Giese RW, Peluso M (2010). Malondialdehyde-deoxyguanosine adduct formation in workers of pathology wards: the role of air formaldehyde exposure. Chem Res Toxicol.

[CR4] Bradford MM (1976). A rapid and sensitive method for the quantitation of microgram quantities of protein utilizing the principle of protein-dye binding. Anal Biochem.

[CR5] Buchmann K, Bresciani J, Jappe C (2004). Effects of formalin treatment on epithelial structure and mucous cell densities in rainbow trout, *Oncorhynchus mykiss* (Walbaum), skin. J Fish Dis.

[CR6] Carvan MJ, Incardona JP, Rise ML (2008). Meeting the challenges of aquatic vertebrate ecotoxicology. Bioscience.

[CR7] Dubinina EE, Burmistrov SO, Khodov DA, Porotov IG (1995). Oxidative modification of human serum proteins. A method of determining it. Vopr Med Khim.

[CR8] Escher BI, Cowan-Ellsberry CE, Dyer S, Embry MR, Erhardt S, Halder M, Kwon JH, Johanning K, Oosterwijk MT, Rutishauser S, Segner H, Nichols J (2011). Protein and lipid binding parameters in rainbow trout (*Oncorhynchus mykiss*) blood and liver fractions to extrapolate from an in vitro metabolic degradation assay to in vivo bioaccumulation potential of hydrophobic organic chemicals. Chem Res Toxicol.

[CR9] FAO Fisheries and Aquaculture Dept. Aquaculture Management and Conservation Service (2007). Improving Penaeus monodon hatchery practices: manual based on experience in India.

[CR10] Francis-Floyd R (1996) Use of formalin to control fish parasites. Document Nr VM-77 of a series of the College of Veterinary Medicine, Florida Cooperative Extension Service, Institute of Food and Agricultural Sciences, University of Florida

[CR11] Friedman SF, Martin P, Munoz JS (2003). Laboratory evaluation of the patient with liver disease. Hepatology, a textbook of liver disease.

[CR12] Gaté L, Paul J, Nguyen Ba G, Tew KD, Tapiero H (1999). Oxidative stress induced in pathologies: the role of antioxidants. Biomed Pharmacother.

[CR13] Gomez JM, Boujard T, Boeuf G, Solari A, Le Bail PY (1997). Individual diurnal plasma profiles of thyroid hormones in rainbow trout (*Oncorhynchus mykiss*) in relation to cortisol, growth hormone, and growth rate. Gen Comp Endocrinol.

[CR14] Granchi C, Bertini S, Macchia M, Minutolo F (2010). Inhibitors of lactate dehydrogenase isoforms and their therapeutic potentials. Curr Med Chem.

[CR15] Güleç M, Songur A, Sahir S, Ozen OA, Sarsilmaz M, Akyol O (2006). Antioxidant enzyme activities and lipid peroxidation products in heart tissue of subacute and subchronic formaldehyde-exposed rats: a preliminary study. Toxicol Ind Health.

[CR16] Gurel A, Coskun O, Armutcu F, Kanter M, Ozen OA (2005). Vitamin E against oxidative damage caused by formaldehyde in frontal cortex and hippocampus: biochemical and histological studies. J Chem Neuroanat.

[CR17] Halliwell B (1994). Free radicals, antioxidants, and human disease: curiosity, cause or consequence?. Lancet.

[CR18] Halliwell B, Gutteridge JMC (1989). Free radicals in medicine and biology.

[CR19] Heck H, Casanova M (1999). Pharmacodynamics of formaldehyde: applications of a model for the arrest of DNA replication by DNA-protein cross-links. Toxicol Appl Pharmacol.

[CR20] Herasimov I, Plaksina O (2000). Non-enzymatic determination of lactate and pyruvate concentrations in blood sample. Laboratorna Diagnostyka.

[CR21] Im H, Oh E, Mun J, Khim JY, Lee E, Kang HS, Kim E, Kim H, Won NH, Kim YH, Jung WW, Sul D (2006). Evaluation of toxicological monitoring markers using proteomic analysis in rats exposed to formaldehyde. J Proteome Res.

[CR22] Kamyshnikov VS (2004). Reference book on clinic and biochemical researches and laboratory diagnostics.

[CR23] Kum C, Kiral F, Sekkin S, Seyrek K, Boyacioglu M (2007). Effects of xylene and formaldehyde inhalation on oxidative stress in adult and developing rats livers. Exp Anim.

[CR24] Lahnsteiner F, Kletzl M (2016). Investigations on the effect of formalin and iodophor on embryo and larvae development in pikeperch, *Sander lucioperca*. J Appl Aquac.

[CR25] Levine RL, Garland D, Oliver CN, Amici A, Climent I, Lenz A-G, Ahn B-W, Shaltiel S, Stadtman ER (1990). Determination of carbonyl content in oxidatively modified proteins. Methods Enzymol.

[CR26] Li H, Wang J, König R, Ansari GA, Khan MF (2007). Formaldehyde-protein conjugate-specific antibodies in rats exposed to formaldehyde. J Toxicol Environ Health A.

[CR27] Li ZH, Li P, Randak T (2011). Evaluating the toxicity of environmental concentrations of waterborne chromium (VI) to a model teleost, *Oncorhynchus mykiss*: a comparative study of in vivo and in vitro. Comp Biochem Physiol C: Toxicol Pharmacol.

[CR28] Lima LF, Murta GL, Bandeira AC, Nardeli CR, Lima WG, Bezerra FS (2015). Short-term exposure to formaldehyde promotes oxidative damage and inflammation in the trachea and diaphragm muscle of adult rats. Ann Anat.

[CR29] Marking LL, Rach JJ, Schreier TM (1994). Evaluation of antifungal against for fish culture. Progress Fish Cultur.

[CR30] Marnett LJ (2002). Oxy radicals, lipid peroxidation and DNA damage. Toxicology.

[CR31] Mommsen TP, Vijayan MM, Moon TW (1999). Cortisol in teleosts: dynamics, mechanisms of action, and metabolic regulation. Rev Fish Biol Fish.

[CR32] Munnia A, Amasio ME, Peluso M (2004). Exocyclic malondialdehyde and aromatic DNA adducts in larynx tissues. Free Radic Biol Med.

[CR33] Munnia A, Bonassi S, Verna A, Quaglia R, Pilucco D, Ceppi M, Neri M, Buratti M, Taioli E, Garte S, Peluso M (2006). Bronchial malondialdehyde DNA adducts, tobacco smoking, and lung cancer. Free Radic Biol Med.

[CR34] Murta GL, Campos KK, Bandeira AC, Diniz MF, de Paula Costa G, Costa DC, Talvani A, Lima WG, Bezerra FS (2016). Oxidative effects on lung inflammatory response in rats exposed to different concentrations of formaldehyde. Environ Pollut.

[CR35] Peluso M, Srivatanakul P, Munnia A, Jedpiyawongse A, Ceppi M, Sangrajrang S, Piro S, Boffetta P (2010). Malondialdehyde-dG adducts among workers of a thai industrial estate and nearby residents. Environ Health Perspect.

[CR36] Persoz C, Achard S, Leleu C, Momas I, Seta N (2010). An in vitro model to evaluate the inflammatory response after gaseous formaldehyde exposure of lung epithelial cells. Toxicol Lett.

[CR37] Reitman S, Frankel S (1957). A colorimetric method for determination of serum oxaloacetic and glutamic pyruvic transaminases. Am J Clin Pathology.

[CR38] Saito Y, Nishio K, Yoshida Y, Niki E (2005). Cytotoxic effect of formaldehyde with free radicals via increment of cellular reactive oxygen species. Toxicology.

[CR39] Sanchez JG, Speare DJ, Sims DE, Johnson GJ (1998). Morphometric assessment of epidermal and mucous-biofilm changes caused by exposure of trout to chloramine-T or formalin treatment. J Comp Pathol.

[CR40] Santovito A, Schilirò T, Castellano S, Cervella P, Bigatti MP, Gilli G, Bono R, DelPero M (2011). Combined analysis of chromosomal aberrations and glutathione S-transferase M1 and T1 polymorphisms in pathologists occupationally exposed to formaldehyde. Arch Toxicol.

[CR41] Sevela M, Tovarek J (1959). A method for estimation of lactic dehydrogenase in body liquids. J Czech Physiol.

[CR42] Shacter E (2000). Quantification and significance of protein oxidation in biological samples. Drug Metab Rev.

[CR43] Shi YQ, Chen X, Dai J, Jiang ZF, Li N, Zhang BY, Zhang ZB (2014). Selenium pretreatment attenuates formaldehyde-induced genotoxicity in A549 cell lines. Toxicol Ind Health.

[CR44] Small BC, Chatakondi N (2006). Efficacy of formalin as an egg disinfectant for improving hybrid catfish (Channel Catfish × Blue Catfish) hatching success. N Am J Aquac.

[CR45] Speare DJ, Arsenault G, MacNair N, Powell MD (1997). Branchial lesions associated with intermittent formalin bath treatment of Atlantic salmon, *Salmo salar* L., and rainbow trout, *Oncorhynchus mykiss* (Walbaum). J Fish Dis.

[CR46] Sul D, Kim H, Oh E, Phark S, Cho E, Choi S, Kang HS, Kim EM, Hwang KW, Jung WW (2007). Gene expression profiling in lung tissues from rats exposed to formaldehyde. Arch Toxicol.

[CR47] Szende B, Tyihák E (2010). Effect of formaldehyde on cell prolifiration and death. Cell Biol Int.

[CR48] Teng S, Beard K, Pourahmad J, Moridani M, Easson E, Poon R, O’Brien PJ (2001). The formaldehyde metabolic detoxification enzyme systems and molecular cytotoxic mechanism in isolated rat hepatocytes. Chem Biol Interact.

[CR49] Thapa BR, Walia A (2007). Liver function tests and their interpretation. Indian J Pediatr.

[CR50] Thorgaard GH, Bailey GS, Williams D, Buhler DR, Kaattari SL, Ristow SS, Hansen JD, Winton JR, Bartholomew JL, Nagler JJ, Walsh PJ, Vijayan MM, Devlin RH, Hardy RW, Overturf KE, Young WP, Robison BD, Rexroad C, Palti Y (2002). Status and opportunities for genomics research with rainbow trout. Comp Biochem Physiol B: Biochem Mol Biol.

[CR51] Thrasher JD, Kilburn KH (2001). Embryo toxicity and teratogenicity of formaldehyde. Arch Environ Health.

[CR52] VandervVeen LA, Hasmin MF, Shyr Y, Marnett LJ (2003). Induction of frame-shift and base pair substitution mutations by the major DNA adduct of the endogenous carcinogen malondialdehyde. P Natl Acad Sci USA.

[CR53] Vijayan MM, Raptis S, Sathiyaa R (2003). Cortisol treatment affects glucocorticoid receptor and glucocorticoid-responsive genes in the liver of rainbow trout. Gen Comp Endocrinol.

[CR54] Wagner EJ, Arndt RE, Billman EJ, Forest A, Cavender W (2008). Comparison of the efficacy of iodine, formalin, salt, and hydrogen peroxide for control of external bacteria on rainbow trout eggs. N Am J Aquac.

[CR55] Williams DE (2012). The rainbow trout liver cancer model: response to environmental chemicals and studies on promotion and chemoprevention. Comp Biochem Physiol C: Toxicol Pharmacol.

[CR56] Yildiz HY, Guzey IM, Ergonul MB (2009). Changes of non-specific immune parameters in rainbow trout, *Oncorhynchus mykiss*, after exposure to antimicrobial agents used in aquaculture. J Appl Aquac.

[CR57] Yu GY, Song XF, Liu Y, Sun ZW (2014). Inhaled formaldehyde induces bone marrow toxicity via oxidative stress in exposed mice. Asian Pac J Cancer Prev.

[CR58] Zar JH (1999). Biostatistical analysis.

[CR59] Zhang BY, Shi YQ, Chen X, Dai J, Jiang ZF, Li N, Zhang ZB (2013). Protective effect of curcumin against formaldehyde-induced genotoxicity in A549 Cell Lines. J Appl Toxicol.

[CR60] Zikić RV, Stajn AS, Pavlović SZ, Ognjanović BI, Saićić ZS (2001). Activities of superoxide dismutase and catalase in erythrocytes and plasma transaminases of goldfish (*Carassius auratus gibelio* Bloch.) exposed to cadmium. Physiol Res.

